# Challenges and coping mechanisms among caregivers of children with orofacial cleft in a Nigerian teaching hospital: photovoice approach

**DOI:** 10.3389/froh.2025.1682928

**Published:** 2025-11-04

**Authors:** Oluwafemi Adeagbo, Oluwaseun Badru, Bolaji Akala, Abimbola Oladayo, Adegbayi Adekunle, Oluwanifemi Ayelomi, Adejoke Babatunde, Olugbemiga Ogunlewe, Olutayo James, Wasiu Lanre Adeyemo, Nnenna Mba-Oduwusi, Azeez Butali

**Affiliations:** 1 Department of Community and Behavioral Health, College of Public Health, University of Iowa, Iowa City, IA, United States; 2 Department of Sociology, Faculty of Humanities, University of Johannesburg Auckland Park, Johannesburg, South Africa; 3 InSiGHt Health Consulting, Ikeja, Lagos, Nigeria; 4 Missouri School of Dentistry and Oral Health, A.T. Still University, Kirksville, MO, United States; 5 Department of Oral and Maxillofacial Surgery, College of Medicine University of Lagos/Lagos University Teaching Hospital, Lagos, Nigeria; 6 Department of Oral Pathology, Radiology and Medicine, College of Dentistry, University of Iowa, Iowa City, IA, United States; 7 Iowa Institute of Oral Health Research, University of Iowa, Iowa City, IA, United States

**Keywords:** orofacial cleft, challenges, photovoice, coping mechanism, caregivers

## Abstract

**Introduction:**

Children with Orofacial cleft (OFC) require specific support that can be challenging for their caregivers due to the various challenges associated with OFC. The use of photovoice in cleft research is scant. Thus, we used the photovoice approach to investigate the challenges and coping mechanisms of caregivers of children with OFC.

**Methods:**

We conducted community-based participatory research using the photovoice approach and focus group discussion to investigate the challenges and coping mechanisms of caregivers (36 females and two males) of children with OFC at the Lagos University Teaching Hospital Cleft Clinic, Idi-Araba, Lagos, Nigeria. Three semi-structured group discussions and one focus group discussion with 10 caregivers across the three groups. Through a day exhibition, the caregivers created awareness about OFC, shared their challenges and coping strategies with caring for a child with OFC to key stakeholders.

**Results:**

The caregivers highlighted the difficulty of caring for children with OFC, particularly feeding and financial constraints. Nasal regurgitation and the inability of children to latch on to the breast made feeding difficult. Most caregivers adopted baby formulas, but they lamented their high cost, exacerbated by either losing their businesses or resigning from work to care for the children. The major coping mechanism was resilience through faith and spirituality.

**Conclusion:**

Burden of care was a major challenge for caregivers of children with OFC. Feeding and financial challenges were major burdens, leading the caregivers to hold on to faith and spirituality for succor. Health providers must educate caregivers on sustainable feeding techniques and management of their ward's feeding to meet the required feeding expectations regarding the children's weight.

## Introduction

Globally, orofacial clefts (OFCs) are the most common birth defects of the head and neck region, affecting 1 in 700 live births (1). In 2019, an estimated 4 million people were affected by OFCs across the world (2). The rate of OFCs varies across ethnic groups and geographic regions, and is lowest in Africans (3). Generally, people living with OFC require comprehensive care ranging from surgical to speech therapy, and the burden of care for clefts often affects the overall quality of life of affected individuals and their families (2, 4, 5). Individuals with clefts, particularly children, may experience behavioral challenges and displeasure with their physical appearance and speech during early childhood development (2, 6, 7). Orofacial clefts often lead to significant financial, educational, medical, psychological, and cultural problems and have been reported to lead to infanticide due to pressures of stigmatization and a lack of sociocultural support for affected families (8–10).

Caring for a child with a cleft requires significant emotional and financial responsibilities from caregivers (2, 8). Growing evidence has shown that caregivers of children with OFC often experience stressors associated with the burden of care (2, 11). Recent studies (including systematic reviews) reported a higher prevalence of mental disorders, such as depression, among caregivers of children with clefts (2, 12). Furthermore, some studies have shown the association between caregivers’ health and that of their children (2, 13). It is therefore imperative to investigate the challenges of care and coping strategies among caregivers of children with OFC. Moreover, individuals respond differently to stigmatizing health conditions like clefts and develop coping mechanisms and resilience (14, 15). Studies in sub-Saharan Africa (including Nigeria) and elsewhere have reported the link between resilience and improved health outcomes among individuals experiencing adversities (14–17).

To identify caregivers’ challenges and coping strategies, we used a participatory research approach called photovoice. Photovoice is an innovative approach to community-based participatory research (CBPR), which uses pictures taken by the participants in addition to storytelling to communicate the participants’ perspectives to critical stakeholders and other people from their community (18–21). A US study conducted among families of children with medical complexities used photovoice approach to identify caregivers’ challenges and concerns as well as their coping strategies to care for their children (19). In photovoice approach, learning occurs via the collaborative efforts of participants in the process of reflecting on and discussing salient community issues that affect them (19, 22). The use of photovoice can lead to an increased awareness of the resources in the community, improved community engagement, and the promotion of self-efficacy among research partners (19, 21, 23). Although previous research has used photovoice to document the perspectives of individuals and communities with varying characteristics, few have used it for caregivers and children working together at all project stages. None of the existing research in resource-constrained settings, such as those found in sub-Saharan Africa (SSA), has focused on or used photovoice to examine the experiences of caregivers of children with OFC, who face unique challenges because of the physical, mental, and psychological needs and associated stigmatization. It is against this backdrop that the current study used photovoice approach to investigate the challenges, concerns, and coping mechanisms of caregivers of children with clefts in Lagos, Nigeria.

## Methods

### Study design and setting

This study adopted a community-based participatory approach (e.g., photovoice) and qualitative design [e.g., focus group discussion (FGD)] to explore caregivers’ experience caring for children with OFC. Specifically, we combined photovoice approach with FGD to understand the challenges of caregivers of children with OFC receiving care at the Lagos University Teaching Hospital (LUTH), Idi-Araba, Lagos State, Nigeria. The tertiary hospital, founded in 1961, serves as a major referral center, including for OFC patients within and outside Nigeria (24).

### Participants and recruitment

The study recruitment and implementation were co-led by InSiGHt Health Consulting, a for-profit local organization with over twenty years of experience implementing health programs and interventions in Nigeria. We purposively recruited caregivers of children with OFC receiving care at LUTH between December 2023 and February 2024. We recruited participants who were at least 18 years old at the time of the study, played a major role in caring for the child, and were available for the study duration. We used the criteria for “not staying” too far to minimize attrition and to increase the likelihood of having sufficient data for analysis. Forty caregivers consented to participate in the study, but two dropped out because they relocated. Therefore, 38 caregivers completed the study, and only two were males. We divided the caregivers into three groups for better coordination and ease of communication.

### Photovoice approach

The study utilized the photovoice method—a community-based participatory research approach—to gather data from caregivers of children with OFC. This method allows participants to tell their own stories. Photovoice originated from the work of Wang & Burris (25). This approach requires that participants, through taking photos, express their lived experiences and views (26). Our study participants were trained on camera use, the photovoice technique as a data collection tool, the basics of photography, and data reporting. Participants were encouraged to take photos that answered the research questions. They described their photos afterwards using the See, Happening, Our lives, Why, Educate, and Do (SHOWeD) method. Through the SHOWeD framework, participants, while speaking to the photos, discuss what they see in a photo, what is happening in the photo, how it affects their daily lives, why the situation in the photo exists, how it can educate or empower the community, and what we can do about the identified issues (26).

#### Photovoice training

We organized a one-day physical training session with the caregivers. As recommended by Wang & Burris (25), the first training was to brief and educate the caregivers on how to use the camera and the ethics of photography. Specifically, we first discussed the study methodology, research questions, camera handling and usage, and the type of photos required. Secondly, we discussed photography ethics to ensure that participants took photos following the right guidelines, including consent seeking when other people or properties are involved in the photographs. Following this, we had practical photography sessions to build participants’ skills and confidence while in the field. Using the pictures taken during the photography session, we further deepen caregivers’ understanding of photography by explaining directions, use of space, shades and light, angles, and framing. We included this to ensure that the participants took high-quality and thought-provoking photos. Finally, we provided and taught the caregivers how to document photographs taken, properly tag them for identification with respect to time, and date from the camera into a pre-designed worksheet.

### Data collection

The entire data collection (FGD, group discussions, and photos) happened between January and May 2024. The caregivers retained the cameras provided for the training for the main project. The caregivers took photos between January and April 2024. To facilitate bidirectional communication between caregivers and the research team, and to provide an opportunity for caregivers to ask questions or discuss challenges promptly during the data collection period, we created three WhatsApp groups (one for each group) with their consent. We called caregivers once a week to gather feedback and address any research-related issues they encountered during the study. Caregivers (*n* = 3) without WhatsApp-enabled mobile phones were called more than once weekly for feedback since they had no access to discussions in their various groups. Furthermore, we held bi-weekly physical check-in meetings with each group separately to address issues and concerns with the data collection. We also reviewed and retrieved photos during those meetings. It was necessary to retrieve photos early because one of the lessons learned in the pretest was the loss of photos, as some participants had mistakenly deleted them. Therefore, we took these steps to preserve the data.

We created a folder for each caregiver with their individual unique IDs and saved the retrieved photos on a secure laptop. We retrieved, sorted, and discussed the photos during the bi-weekly meeting with each group. In each group (*n* = 3 group discussions), the caregivers discussed their photos using the SHOWeD framework, and the group selected representative photos. Later, all groups met in a combined group meeting to discuss and analyze the 75 photos chosen across the groups. The team asked caregivers whose photos were selected to discuss their photos using the SHOWeD approach, and the group collectively chose the final exhibition photos. They selected 30 photos (including a narrative for each photo) that best represented their experiences and presented them in an exhibition to key stakeholders such as cleft care providers, government officials, non-governmental organizations, community members, and researchers. Furthermore, six weeks after the end of the photovoice activities, we conducted one FGD with 10 participants across the three groups in May 2024 to further understand their experiences (including challenges and coping strategies) with caring for children with OFC, as well as taking photos to document their challenges and happy moments. An experienced male interviewer (research assistant) led the group discussions and the FGD, while another assistant was responsible for notetaking. The interviews were audio-recorded with the consent of the participants. The group discussions and the FGD lasted between 90 min and 180 min, respectively, and were conducted in English. Besides the light refreshments we provided, each participant received N15,000 per visit (US$10) and was asked to keep the study camera at the end of the study as a token of appreciation for their time and participation.

### Photo exhibition

The photo exhibition was held on July 3, 2024, at LUTH, with the following stakeholders in attendance: the caregivers, dental consultants, past and present LUTH Medical Directors and the College Provosts, Smile Train team, InSiGHt Health Consulting team, the University of Iowa research team, journalists, and the media.

The exhibition was in four parts. First, a brief overview of the project was shared with all present at the exhibition. Second, we created a 7-minute video documentary based on our findings, which prompted a dialogue with the audience. Third, five representative caregivers nominated by their colleagues shared their experiences with the stakeholders, guests, and other caregivers who were present. This was followed by a discussion. Finally, we displayed 15 boards, each containing two photograph posters with illustrations that addressed the project objectives and research questions. The caregivers, whose photographs were displayed, then interacted with stakeholders who visited their respective boards, shedding more light on their pictures, i.e., why they took those pictures and what they meant to them.

### Data management and analysis

An experienced transcriber manually transcribed all audio files verbatim into texts, and the transcripts were checked for accuracy by another interviewer. All identifying information was removed from the interview transcripts to protect confidentiality. The transcripts were checked with the audio files to make sure participants’ thoughts were captured correctly. The data was stored and managed in a secure web-based shared drive with restricted access.

Two researchers (OB, BA) skilled in qualitative methods manually coded the interview transcripts. Following the Braun & Clarke approach, we adopted inductive thematic analysis for the photo narratives and interview transcripts (27). The approaches included (1) familiarization with the findings, (2) initial coding, (3) identification of themes, (4) extensive review of the themes, (5) theme and sub-theme definition, and (6) finding report (27). The analysis was conducted by an experienced PhD student (OB) and one of the investigators (OA), who has over 15 years’ experience in qualitative research and implementation science. The two analysts (OA & OB) independently coded and compared coding until consensus was reached; the same procedure was carried out to ascertain inter-coder reliability. The analysts reiteratively assessed and selected the representative quotes and photos without bias. Moreover, the analysts shared the selected quotes and photos with the broader research team for further validation.

To ensure rigor, we followed the eight criteria for excellent qualitative research by Tracy (28) as well as the consolidated criteria for reporting qualitative research (COREQ) checklist (29). First, we ensure self-reflexivity by constantly checking our biases as researchers and providing a detailed account of our methodology. Second, we ensured credibility by providing a detailed description of the methodology, enabling reproducibility and transferability, and triangulated findings from the FGD with those from the photovoice analysis for a more robust finding. Lastly, we ensured meaningful coherence by connecting our findings and situating them in line with earlier studies.

## Results

### Participant characteristics

Most of the caregivers were females (36; 95%) and mostly mothers (35; 92.1%) between 31 and 40 years old (19; 50.0%) and had tertiary-level education (23; 60.5%; Table 1). Notably, we did not see any differences in themes by age group or gender.

**Table 1:** Sociodemographic characteristics of the caregivers (*n* = 38).

Sociodemographic	Frequency	Percentage
Sex		
Female	36	94.7
Male	2	5.3
Age (years)		
21–30	7	18.4
31–40	19	50.0
41–50	12	31.6
Highest level of education		
Primary	5	13.2
Secondary	10	26.3
Tertiary	23	60.5
Relationship with child		
Mother	35	92.1
Father	2	5.3
Grandmother	1	2.6

### Key findings from the qualitative analysis

When we asked the caregivers what has made it easier or harder to live their daily lives as caregivers of a child with OFC and to be part of their community, challenges such as caring for children with OFC, feeding difficulty, financial challenges and expensive baby food were common; and they identified faith and spirituality to be their key coping strategies.

### Theme 1: challenges associated with caring for children with OFC

Participants highlighted some of the challenges associated with taking care of their children with OFC, as identified below.

#### The burden of care

The burden of care among participants is multifaceted. All the caregivers clearly expressed that caring for a child with OFC is a difficult task.

A participant shared their frustrations of taking care of two children simultaneously:

“Caring for a patient with clefts, especially when the babies were twins, like mine, wasn't easy… even at night, sometimes, when they were babies, the other would cry. I would have to take care of the twin sister with clefts, especially when I put the appliance in her mouth. It was not easy at all” (P1).

Several caregivers reported that it was difficult for them to present the children in the community due to stigmatization and discrimination, leading to the constant need to hide their children. One participant captures this better:

“To me, I am not happy, so I had to hide the child from other people, for them not to see her and gossip about it in the streets. It is not easy for me to have a baby you cannot carry… you cannot show them off, you have to keep them separate” (P2).

Some caregivers also mentioned that their movement was restricted and leaving the children for a longer period was difficult because they needed closer monitoring and special care:

“It was not easy living with a cleft or palate child because you cannot move around easily the way you used to do. But when you are going anywhere your mind will be on that child. How is he feeling?” (P5).

Furthermore, some participants mentioned that the burden of care resulted from resigning or stopping work to care for their children full-time. This led to lower earning power and socioeconomic status. A participant explained this challenge concisely:

“I have to ask myself: do I want to take to my career, or do I want to stay with this child? She needs me at this moment in her life. You know, I have to give up my job. At the time I was resigning, I was crying that God, it is not as if it is intentional for me to resign, it is not as if they sacked me, but I have to sacrifice my own time, you know, my career just because I want to take care of this girl, God, please support me, help me on this journey..” (P5).

#### Feeding difficulty

During the FGDs, many of the caregivers overwhelmingly articulated that feeding was a major challenge in caring for children with OFC. For example, one caregiver mentioned her ordeal with nasal regurgitation while feeding her child:

“There was an experience I had..I thought, after feeding her, let me just put her down immediately I placed her down she had to, you know, bring everything out from her nose, her mouth” (P2). In fact, some caregivers mentioned that allowing an “inexperienced” person to feed their children was not recommended: “Feeding them [children with cleft] is also not easy. You cannot give them to an ordinary person to feed them for you” (P4).

**Figure 1:**
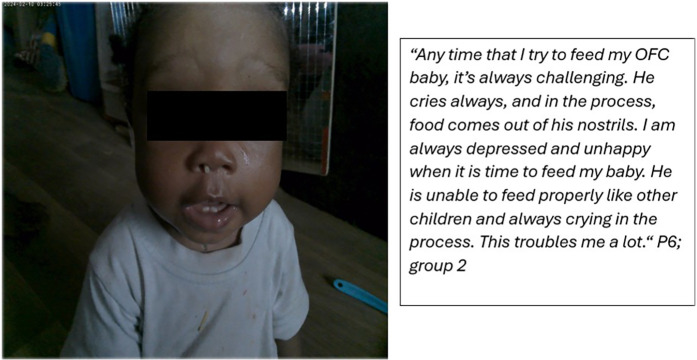
Feeding is an emotionally distressing experience for mothers of children with OFC.

Similarly, speaking to one of her photos (Figure 1), a caregiver narrated how “*food comes out of…nostrils.” (P6; group meeting 2)*.

We probed how caregivers resolved this feeding difficulty, and most of them had an improvised spoon that enabled them to feed the children:

“…so there is this kind of spoon, not really spoon, like a feeder wait is like a spoon form, yes, that was given to us. So, it really helped me a lot in feeding her” (P2).

Moreover, many caregivers mentioned the breastfeeding challenge in particular as one of the difficulties of caring for children with OFC:

“The first challenge was breastfeeding because I have a baby with a bilateral cleft lip and palate, so it's not really easy for him to breastfeed to suck” (P1).

Additionally, the caregivers hinted that feeding difficulty was bidirectional—the inability of the infants to suck or latch was reported earlier as poor milk supply from the breast. We probed how caregivers overcame this particular feeding challenge; most relied on breast pumps. For example, one caregiver said:

“I went to go and buy the electric one that I'm using to breast pump it so that that one is now easier” (P5).

### Financial constraint

Financial struggle was another major challenge that emerged. Many caregivers struggled with the financial need of caring for the children. This includes funds needed to buy baby formulas, run medical tests, and perform the much-needed surgeries. For example, following the OFC diagnosis, one caregiver was concerned about the money required to care for the child:

“Then, after we have been told the process of bringing the child back to normal, it boils down to financial challenges…I remember when I have the thought of how do I raise money for so-so test so-so this so-so that it was a real challenge” (P4).

**Figure 2:**
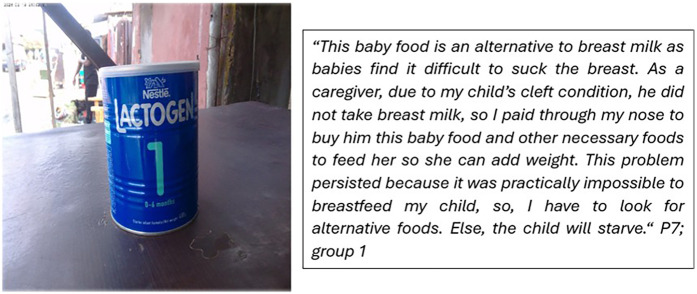
The financial impact of OFC on families.

While interpreting their photos on feeding difficulty, some caregivers lamented about the cost of baby formula (Figure 2). Below is a representative photo and a description of the feeding challenge they experienced:

Another caregiver narrated personal sacrifices they had to make alongside financial support from family members before they could buy baby formula to feed their child:

“…when I pump now, in the next 30 min, she wants to eat again. If I want to pump again, there is no breast milk again. I have to complain. They said I should go and buy NAN One [baby formula]. Even when I have not eaten enough, I have to go and buy another one. Where will I get money for another one? I will start begging. I will call my brother, hello. I will be calling them; please help me. Somebody will send N5,000 today; I will buy [baby formula]. Before that one finishes, I will still call another person. Two, three days, that's finished. I was ashamed of myself…I only stay at home with her. And there is no way to get the money…” (P5).

Furthermore, some participants mentioned that the burden of care exacerbated their financial struggles, as some of them had to sacrifice jobs that could put them in a better financial position to care for their children. A participant shared her experience:

“…when this baby came, I was at the tip of grabbing something very big [employment opportunity], but I had to drop it, so the fact that I had to drop so many things that will bring me very great, great achievements..I don't joke with business. I don't joke with anything that is supposed to push me to the next [level], but…everybody around me…was surprised that…I had to drop those things” (P4).

We asked the caregivers about the ways they navigated their financial struggles, and many of them mentioned how they got surgical support through Smile Train (an international non-governmental organization providing financial support for cleft surgery):

“…to the glory of God, he brought Smile Train to us because if not, I remember the first surgery I did for my son. I don't know about Smile Train. I knew what it took me and the family to come to have that surgery done and the stress involved. I so much give it to this Smile Train unit in LUTH. They are extraordinary because I've done [first child surgery], I did my first son. I have two children with clefts. I did my first son own the first one outside LUTH… I knew what I passed through, so Smile Train came and gave us a platform to breathe because before we saw personally, this is my personal experience…” (P3).

One participant narrated the immediate impact of the free surgery on caring for her child:

“I overcome through Smile Train, especially the challenge of bathing. First day we got home after the surgery, I bathed my baby from [the] head like I poured water over her head… I bathed her from the head; nothing happened. Smile Train is the secret to my smile” (P4).

### Theme 2: resilience through faith and spirituality

#### Faith and spirituality

**Figure 3:**
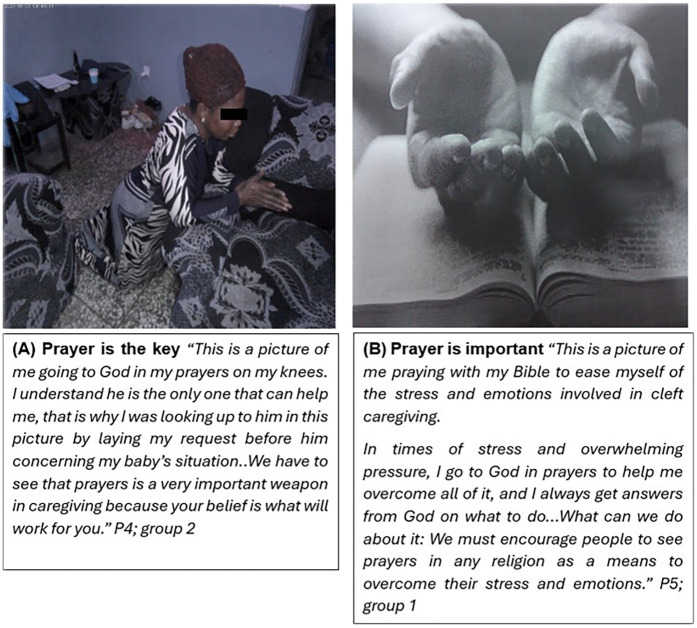
Prayer is an improtant coping mechanism for mothers of OFC infants.

Group discussion transcripts, along with several photos taken by participants and the reasons behind them, point to the fact that caregivers sought resilience in faith and spirituality. Some of them alluded to their belief in the supreme being and prayers. For example, in Figures 3A,B, some participants describe how they pray to relieve challenges, pressure, and stress associated with caring for a child with a cleft.

Similarly, in the FGD, the caregivers affirmed that spirituality, faith, and hope made them resilient to some of the challenges they faced caring for their children with OFC. Prayer and faith in God were their coping strategies as reported by the following participant: “*…these issues make me to know that I'm a strong woman. I can overcome anything with God”* (P3):

Another participant interpreted her caring for a child with cleft as a test from God, and she believed being closer to God through her faith and in prayer has helped her thus far:

“God can test anybody in any way, so don't think I am too religious, I am too smart, I am this, I am that. No, the test of God comes in a…and we need to take it with pride and face it up and God wants to know how best you can do or if you are going to pass it or not, so it is a kind of test to me, and to God be the glory, we have been able to do it. And the last one is that it makes me more closer to God…I commit everything [to God]; God I am going to LUTH today let me go and come back safe, this surgery I want to do. I commit everything into your hands, and He has been there for me” (P5).

## Discussion

We explored the challenges OFC caregivers face and the strategies they have used to foster resilience with photovoice approach and FGDs. Both FGD transcripts and photo illustrations show that caring for children with OFC was challenging for our study participants, particularly the issues around feeding. This finding is consistent with the previous literature (30, 31). For example, caregivers in Kenya (31) and South Africa (30) have also reported feeding difficulty as the biggest challenge they face in caring for children with OFC. One common feeding problem was the nasal regurgitation of ingested food, similar to what other studies have reported (30, 32). Ligot and colleagues (32), for example, reported that more than half of the caregivers they interviewed in the Philippines experienced feeding challenges with their children. Most of the caregivers we interviewed find feeding challenging due to nasal regurgitation, and they addressed this issue by using specialized spoons to feed their children. Caregivers of children with clefts in Kenya, Uganda, and the Philippines have also reported using specialized spoons and other techniques, such as syringes and softplas feeding bottles, to address their children's feeding difficulties (31–33).

In addition to nasal regurgitation, female caregivers highlighted that their feeding challenge was sometimes due to a lack of breast milk production. This echoes the findings of Kaye et al. (34) who found that 90% of mothers who stopped breastfeeding did so because of loss of breast milk production. Moreover, we found that mothers used breast pumps, similar to some earlier reports (33, 34), to sustain breast milk production and to overcome breastfeeding difficulties. However, some mothers in Uganda reported that breast pumps did not sustain milk production (33). The possible explanation for the lack of sustained milk production could be the inadequate stimulation of the hormones (prolactin and oxytocin) needed to sustain breast milk production via infant suckling (35).

The feeding difficulties these caregivers face have implications for them and their children. For example, while spoons and other feeding techniques can alleviate the feeding challenge for children with OFC to some extent, there is evidence that they can experience more parental stress than mothers who breastfeed (36). Furthermore, poor latching or prolonged need to feed the children can be tiring, leading to feeding with a less required volume, which is associated with weight loss and, in some cases, malnutrition or death (34, 36, 37). Ligot et al. (32) concluded that feeding difficulty ideally should not be a challenge, at least for the nasal regurgitation, with the modified feeding bottles (e.g., infant- and caregiver-directed bottles) (38) and positioning techniques available that can be taught to caregivers, especially breastfeeding mothers, to avoid nasal regurgitation (32). We recommend that hospital facilities providing care to children with OFC may need to liaise with non-governmental organizations like Smile Train to subsidize supplementary milk and specialized bottles, such as softpals, to relieve caregivers of their feeding difficulties.

We also observed that many caregivers in our study struggled financially while caring for their children. The caregivers discussed the cost of baby formula and medical bills, such as surgery. However, unlike the reports from other studies that the cost of surgery was a major financial struggle and even the biggest challenge in caring for children with OFC (39, 40). Our study participants complained and emphasized the financial cost of feeding more than OFC surgery, perhaps because Smile Train takes care of surgical costs at the cleft hospital. Specifically, many caregivers in our study complained about the cost of purchasing supplementary milk (formula) to feed their children and the specialized feeding bottles.

This issue has also been raised by other caregivers in Egypt (41), Uganda (33), Kenya (31), and Iran (42). Raising money to ensure that their children were fed well was sometimes exacerbated by the loss of jobs or inability to work or earn money, with many caregivers citing the need to sacrifice their jobs to provide more comprehensive and specialized care to the children. Loss of socioeconomic power associated with the burden of care, at least for many mothers, appears to be common among caregivers in similar settings. For instance, some mothers in Kenya (31) and South Africa (30) reported the need to quit their jobs or not get paid because of frequent absences from work to care for their children. This calls for employers to support caregivers of children with OFC to alleviate the financial, emotional, and psychological stress of caring for their children.

To manage the stress associated with caring for their children (especially feeding and the financial needs), many participants opted to be more spiritual and faithful in their relationship with God to foster resilience, believing their children would get the needed help and lead a normal life like children without clefts. Some caregivers in our study considered having a child with OFC as their fate and a test from God. This is similar to what Alinezhad et al. (42) found in their study with some of their participants accepting having a child with a cleft as “destiny.” Similarly, caregivers in Brazil have used religion and spirituality as a way to cope with the parenting stress associated with caring for children with OFC (43). Farinha and colleagues found that despite the feeding and parenting stress, caregivers with positive religious beliefs coped better than their counterparts who showed negative religious beliefs (43). Using religion as a coping mechanism is not uncommon, and many religious scholars have theorized that this is a common coping mechanism, especially in religious societies. For example, the Pargament's Theory of Religious Coping, developed by Kenneth Pargament (44), posits that in critical health situations, when there is a search for significance in times of stress, spirituality is often one of the critical coping mechanisms leveraged.

Yuan et al.'s (45) findings may partly explain why caregivers with spiritual beliefs may be more resilient than others. They reported from their disaggregated linear regression that hope was the only variable significant for mothers and fathers, which may be associated with their spiritual beliefs (45). In other words, caregivers who place their hopes on God as a coping mechanism may show better resilience as they navigate the challenges of caring for children with OFC. However, resilience through spirituality may be one-sided because more than 90% of the caregivers we interviewed were mothers or grandmothers. Therefore, future studies may need to engage an equal or greater number of male caregivers to achieve a balanced view and a robust comparison.

## Limitations and strengths

This study has some limitations and strengths. This study uniquely combined the photovoice approach and FGDs to investigate cleft lip and palate. This adds nuance to our understanding of various intersecting challenges faced by caregivers of children with clefts and how they cope with those challenges. Although we trained and encouraged caregivers to take photos representing the challenges they face caring for children with OFC and their coping strategies, their lack of expertise in photography may have resulted in a lack of more representative images. Additionally, the caregivers' experiences may be skewed because most were mothers, and the financial challenges narrated may be incomplete because fathers (*n* = 2/38), the minority in our study, often play more financial roles than mothers in the research setting. We believe the fathers would have provided more in-depth information regarding the economic difficulties the family experiences as the “breadwinner” in an African setting. This is particularly important because there is evidence that fathers of children with OFC had a higher level of resilience than mothers (45). Furthermore, the faith-based coping mechanism adopted by the caregivers may be a cultural reflection of their society and may not apply to other settings. As such, this finding should be interpreted with caution. Finally, the generalization of findings may be limited to the population and research setting.

## Conclusion

Caregivers in our study predominantly face challenges caring for their children with OFC, mainly feeding difficulties and financial stress, but they use faith and spirituality to foster resilience. Our findings indicate that caregivers need financial support for surgery, baby formula, and access to feeding equipment. Furthermore, health professionals must educate caregivers on how to feed and help them manage their expectations regarding feeding and the child's body weight. Additionally, it will be beneficial for caregivers if health professionals can provide counselling on the overall care of children with OFC to improve their overall well-being. We opine that prenatal OFC may be the best time to commence counseling because they may better manage expectations and be more psychologically ready to care for their child. Also, future research should include more male caregivers to get nuanced opinions on their specific challenges (particularly financial constraints) and coping strategies. Finally, future research should explore how stigma from families, social networks, and the broader community may contribute to the difficulties and stresses encountered by caregivers of children with OFC.

## Data Availability

The data analyzed for this manuscript are not publicly available. However, the corresponding author can make the data available upon reasonable request.

## References

[1] Ma Q Wei J Peng B Liu J Mo S. Burden of orofacial clefts from 1990 to 2021 at global, regional, and national levels. Front Pediatr. (2025) 13:1502877. 10.3389/fped.2025.1502877 40191646 PMC11968431

[2] Oladayo AM Sule V Oshodi Y Adekunle AA Adeyemo WL Ogunlewe O Assessing the psychosocial impacts of whole-genome sequencing outcomes on orofacial cleft caregivers in Nigeria: a mixed-methods study. Cleft Palate Craniofac J. (2025) 0(0):10556656251332351. 10.1177/10556656251332351 40223298

[3] Butali A Adeyemo WL Mossey PA Olasoji HO Onah II Adebola A Prevalence of orofacial clefts in Nigeria. Cleft Palate Craniofac J. (2014) 51:320–5. 10.1597/12-135 23557093 PMC3706513

[4] Awoyale T Onajole AT Ogunnowo BE Adeyemo WL Wanyonyi KL Butali A. Quality of life of family caregivers of children with orofacial clefts in Nigeria: a mixed-method study. Oral Dis. (2016) 22:116–22. 10.1111/odi.12379 26456150 PMC4744119

[5] Galloway J Davies G Mossey PA. International knowledge of direct costs of cleft lip and palate treatment. Arch Pediatr Surg. (2017) 1:10–25. 10.36959/472/347

[6] Murray L Arteche A Bingley C Hentges F Bishop DVM Dalton L The effect of cleft lip on socio-emotional functioning in school-aged children. J Child Psychol Psychiatry. (2010) 51:94–103. 10.1111/j.1469-7610.2009.02186.x 19968739

[7] Waylen A Mahmoud O Wills AK Sell D Sandy JR Ness AR. Centre-level variation in behaviour and the predictors of behaviour in 5-year-old children with non-syndromic unilateral cleft lip: the cleft care UK study. Part 5. Orthod Craniofac Res. (2017) 20:40–7. 10.1111/ocr.12187 28661083 PMC5836977

[8] Stewart BT Hatcher KW Sengupta A Burg RV. Cleft-Related infanticide and abandonment: a systematic review of the academic and lay literature. Cleft Palate Craniofac J. (2018) 55:98–104. 10.1177/1055665617721919 34162058

[9] Adetayo O Ford R Martin M. Africa has unique and urgent barriers to cleft care: lessons from practitioners at the pan-African congress on cleft lip and palate. Pan Afr Med J. (2012) 12:15. 10.11604/pamj.2012.12.15.1638 22826739 PMC3396861

[10] Oginni FO Asuku ME Oladele AO Obuekwe ON Nnabuko RE. Knowledge and cultural beliefs about the etiology and management of orofacial clefts in Nigeria’s major ethnic groups. Cleft Palate Craniofac J. (2010) 47:327–34. 10.1597/07-085.1 20590456

[11] Gbolahan OO Amiede OS Samuel OA. The burden and perceived stress on family caregivers of patients with orofacial cleft deformities in the perioperative period of cleft repair. J Patient Exp. (2020) 7:1602–9. 10.1177/2374373520948650 33457620 PMC7786686

[12] Namdar P Pourasghar M Alizadeh FL Shiva A. Anxiety, depression, and quality of life in caregivers of children with cleft lip and palate: a systematic review. Iran J Psychiatry Behav Sci. (2022) 16(2):1–14. 10.5812/ijpbs-113591

[13] Panter-Brick C Grimon M-P Eggerman M. Caregiver-child mental health: a prospective study in conflict and refugee settings. J Child Psychol Psychiatry. (2014) 55:313–27. 10.1111/jcpp.12167 24286507

[14] Ueki S Fujita Y Kitao M Kumagai Y Ike M Niinomi K Resilience and difficulties of parents of children with a cleft lip and palate. Jpn J Nurs Sci. (2019) 16:232–7. 10.1111/jjns.12231 30155974

[15] Yusof MS Ibrahim HM. Delineating resilience in children with cleft lip and palate (CL/P): a cross-sectional study. Egypt J Otolaryngol. (2024) 40:168. 10.1186/s43163-024-00717-y

[16] Adamu A Mchunu G Naidoo JR. Stress and resilience among women living with HIV in Nigeria. Afr J Prim Health Care Fam Med. (2019) 11:2046. 10.4102/phcfm.v11i1.2046 31714123 PMC6852334

[17] Hussen SA Tsegaye M Argaw MG Andes K Gilliard D del Rio C. Spirituality, social capital and service: factors promoting resilience among expert patients living with HIV in Ethiopia. Glob Public Health. (2014) 9:286–98. 10.1080/17441692.2014.880501 24520996 PMC4033693

[18] Lechuga-Peña S Mitchell FM Poola C Gutiérrez M Rivera LA. Incorporating photovoice into a community-based intervention with latinx families: lessons learned from your family, your neighborhood. Adv Soc Work. (2021) 21:1124–40. 10.18060/24385

[19] Sonsteng-Person M García-Pérez J Copeland V Liévano-Karim L Abrams D Jarman B What I would do to take away your pain”: a photovoice project conducted by mothers of children with medical complexity. Qual Health Res. (2023) 33:204–19. 10.1177/10497323221146047 36704955

[20] van Hees S Horstman K Jansen M Ruwaard D. Photovoicing the neighbourhood: understanding the situated meaning of intangible places for ageing-in-place. Health Place. (2017) 48:11–9. 10.1016/j.healthplace.2017.08.007 28889043

[21] Yahemba D Chowdhury S Olorunfemi T Dubukumah L David A Umunnakwe C Exploring the impact of COVID-19 on frontline health workers through a photovoice study in Kaduna, Kwara and Ogun states, Nigeria. Int Health. (2023) 15:i110–25. 10.1093/inthealth/ihad005 36960811 PMC10037267

[22] Budig K Diez J Conde P Sastre M Hernán M Franco M. Photovoice and empowerment: evaluating the transformative potential of a participatory action research project. BMC Public Health. (2018) 18:432. 10.1186/s12889-018-5335-7 29609576 PMC5879794

[23] Israel BA Coombe CM Cheezum RR Schulz AJ McGranaghan RJ Lichtenstein R Community-based participatory research: a capacity-building approach for policy advocacy aimed at eliminating health disparities. Am J Public Health. (2010) 100:2094–102. 10.2105/AJPH.2009.170506 20864728 PMC2951933

[24] LUTH. Lagos University Teaching Hospital. (2025). Available online at: https://luth.gov.ng/about-us/history/ (Accessed March 5, 2025).

[25] Wang C Burris MA. Photovoice: concept, methodology, and use for participatory needs assessment. Health Educ Behav. (1997) 24:369–87. 10.1177/109019819702400309 9158980

[26] Connors JDN Conley MJ Lorenz LS. Use of photovoice to engage stakeholders in planning for patient-centered outcomes research. Res Involv Engagem. (2019) 5:39. 10.1186/s40900-019-0166-y 31908846 PMC6939291

[27] Braun V Clarke V. Using thematic analysis in psychology. Qual Res Psychol. (2006) 3:77–101. 10.1191/1478088706QP063OA

[28] Tracy SJ. Qualitative quality: eight “big-tent” criteria for excellent qualitative research. Qual Inq. (2010) 16:837–51. 10.1177/1077800410383121

[29] Tong A Sainsbury P Craig J. Consolidated criteria for reporting qualitative research (COREQ): a 32-item checklist for interviews and focus groups. Int J Qual Health Care. (2007) 19:349–57. 10.1093/intqhc/mzm042 17872937

[30] Hlongwa P Rispel LC. People look and ask lots of questions”: caregivers’ perceptions of healthcare provision and support for children born with cleft lip and palate. BMC Public Health. (2018) 18:506. 10.1186/s12889-018-5421-x 29661170 PMC5902984

[31] Kimotho SG Macharia FN. Social stigma and cultural beliefs associated with cleft lip and/or palate: parental perceptions of their experience in Kenya. Humanit Soc Sci Commun. (2020) 7:1–9. 10.1057/s41599-020-00677-7

[32] Ligot FAC Bautista PEC Bunyi KMG. A qualitative study on the feeding methods of Filipino mothers of children with cleft lip and palate aged 0 to 24 months: a pilot study. Acta Med Philipp. (2024) 58(3):23–33. 10.47895/amp.vi0.6625 38966839 PMC11219549

[33] Nabatanzi M Seruwagi GK Tushemerirwe FB Atuyambe L Lubogo D. Mine did not breastfeed”, mothers’ experiences in breastfeeding children aged 0 to 24 months with oral clefts in Uganda. BMC Pregnancy Childbirth. (2021) 21:100. 10.1186/s12884-021-03581-3 33516176 PMC7847043

[34] Kaye A Cattaneo C Huff HM Staggs VS. A pilot study of mothers’ breastfeeding experiences in infants with cleft lip and/or palate. Adv Neonatal Care. (2019) 19:127. 10.1097/ANC.0000000000000551 30325751

[35] Canul-Medina G Fernandez-Mejia C. Morphological, hormonal, and molecular changes in different maternal tissues during lactation and post-lactation. J Physiol Sci. (2019) 69:825–35. 10.1007/s12576-019-00714-4 31564033 PMC10717399

[36] Boztepe H Çınar S Fatma Figen ÖM. Parenting stress in turkish mothers of infants with cleft lip and/or palate. Cleft Palate Craniofac J. (2020) 57:753–61. 10.1177/1055665619898592 31950852

[37] Martin V Greatrex-White S. An evaluation of factors influencing feeding in babies with a cleft palate with and without a cleft lip. J Child Health Care. (2014) 18:72–83. 10.1177/1367493512473853 23439590

[38] Chee-Williams JL Kotlarek K. A Tutorial for Feeding Infants With Orofacial Clefting: General Guidelines and Patient-Specific Intervention. Rockville, MD: Perspectives of the ASHA Special Interest Groups (2024). 10.1044/2024_PERSP-24-00179

[39] Hasanzadeh N Khoda MO Jahanbin A Vatankhah M. Coping strategies and psychological distress among mothers of patients with nonsyndromic cleft lip and palate and the family impact of this disorder. J Craniofac Surg. (2014) 25:441. 10.1097/SCS.0000000000000483 24481167

[40] Plonkowski AT Naidu P Davis GL Etemad S Otobo DD Dwyer AM Barriers to timely primary cleft surgery in patients treated by an international cleft-focused NGO across 18 countries. World J Surg. (2025) 49(3):664–74. 10.1002/wjs.12469 39961773

[41] Morsi AO Yehia AM Badran AS Khattab NMA. Challenges and concerns faced by parents of a group of Egyptian children with cleft lip/palate: a qualitative study. BMC Oral Health. (2023) 23:1011. 10.1186/s12903-023-03747-9 38104058 PMC10725599

[42] Alinezhad D Mohammadi F Kharazifared MJ Gholami M Sarmadi S Razeghi S. Parents’ views and experiences of raising babies born with cleft lip and palate: a qualitative study. BMC Pediatr. (2025) 25:33. 10.1186/s12887-024-05379-6 39815243 PMC11734495

[43] Farinha FT Bom GC Manso MMFG Prado PC Matiole CR. dos Santos Trettene A. Religious/spiritual coping in informal caregivers of children with cleft lip and/or dysphagic palate. Rev Bras Enferm. (2021) 75:e20201300. 10.1590/0034-7167-2020-1300 34787276

[44] Pargament KI. The Psychology of Religion and Coping: Theory, Research, Practice. New York: The Guilford Press (1997). p. 548. Available online at: https://is.muni.cz/el/phil/jaro2008/RLB246/um/K._I._Pargament_Chapters_2___7.pdf

[45] Yuan L Gao Y Pan B Wang J Wang Y Gong C Resilience and related factors: a comparison of fathers and mothers of patients with cleft lip and/or palate in China. Front Psychiatry. (2022) 12:article number 791555, pages 1–12. 10.3389/fpsyt.2021.791555 PMC8792896 35095604

